# Latest Evidence on Intravascular Imaging: A Literature Review

**DOI:** 10.3390/jcm14134714

**Published:** 2025-07-03

**Authors:** Rafail Koros, Antonios Karanasos, Michail I. Papafaklis, Georgia Xygka, Georgios Vasilagkos, Anastasios Apostolos, Fotios Kallinikos, Maria Papageorgiou, Nikoletta-Maria Tampaki, Charikleia-Maria Fotopoulou, Eleni Lolou, Georgia Gkioni, Periklis Davlouros, Grigorios Tsigkas

**Affiliations:** 1 Cardiology Department, University Hospital of Patras, 26504 Patras, Greece; akaranasos@hotmail.com (A.K.); m.papafaklis@yahoo.com (M.I.P.); georgia.xiga@gmail.com (G.X.); george.vasilagkos@hotmail.com (G.V.); anastasisapostolos@gmail.com (A.A.); fotiskallinikos@gmail.com (F.K.); maria.th.papageorgiou@gmail.com (M.P.); ntampaki.nt@gmail.com (N.-M.T.); fotopoulouchara@gmail.com (C.-M.F.); ellolou19@gmail.com (E.L.); geogkion@gmail.com (G.G.); pdav@upatras.gr (P.D.); gregtsig@upatras.gr (G.T.); 2 Faculty of Medicine, University of Patras, 26504 Rio, Greece

**Keywords:** intravascular imaging, percutaneous coronary intervention, intravascular ultrasound, optical coherence tomography, stent optimization, complex coronary lesions

## Abstract

Intravascular imaging (IVI) has emerged as a pivotal tool in percutaneous coronary intervention (PCI), offering superior visualization of coronary anatomy compared with conventional angiography. This literature review synthesizes the latest evidence from randomized trials and meta-analyses published since 2022, assessing the comparative efficacy of IVI modalities—including intravascular ultrasound (IVUS) and optical coherence tomography (OCT)—in complex coronary lesions. Multiple landmark trials, such as RENOVATE-COMPLEX PCI, ILUMIEN IV, OCTOBER, and OCTIVUS, demonstrated that IVI-guided PCI significantly improves procedural outcomes, stent optimization, and clinical endpoints such as target-vessel failure, myocardial infarction, and stent thrombosis. OCT was shown to be particularly beneficial in bifurcation and left main interventions, while IVUS consistently improved outcomes in long lesions and complex anatomies. Despite some trials not meeting their primary clinical endpoints, substudy findings and pooled analyses support a shift toward routine IVI use in anatomically complex cases. Consequently, updated guidelines now recommend IVI as a Class I indication in select patient populations. These findings underscore the need for broader clinical adoption and training in IVI techniques to enhance PCI outcomes.

## 1. Introduction

Coronary angiography remains the most widely used method for guiding coronary angioplasty and assessing the severity and extent of coronary artery disease. It visualizes the vessel lumen using contrast but provides no information about plaque morphology or the specific characteristics of the culprit lesion. Intravascular imaging (IVI) has emerged as a valuable adjunct to angiography, offering detailed assessment of plaque structure and enabling more precise optimization of percutaneous coronary intervention (PCI). This is reflected in the most recent ESC guidelines on Chronic Coronary Syndromes, which assign a Class I recommendation to IVI use in anatomically complex lesions—particularly those involving the left main stem, true bifurcations, or long segments. Compared with angiography, IVI is superior for detecting calcium and evaluating lesion eccentricity, leading to more accurate assessment of both left main and non-left main lesions. IVI modalities, such as intravascular ultrasound (IVUS) and optical coherence tomography (OCT), are well-established techniques that play a critical role in optimizing stent implantation outcomes. By providing high-resolution, cross-sectional visualization of the vessel lumen and stent architecture, these modalities enable precise detection of stent malapposition, underexpansion, and other suboptimal deployment characteristics. Their use has been associated with a reduction in stent-related adverse events, thereby contributing to improved procedural safety and long-term clinical outcomes. IVI plays a role throughout PCI—from pre-procedural lesion assessment and stent sizing, to procedural guidance and post-deployment optimization. Additionally, in acute coronary syndromes, IVI can help identify the culprit lesion and its underlying mechanism—such as plaque rupture, erosion, or less common causes like dissection [1,2,3].

### 1.1. Principles of Intravascular Imaging

Intravascular ultrasound images are generated when a piezoelectric transducer (crystal) moves in oscillatory motion. The current that passes through the piezoelectric material expands and contracts, producing sound waves when electrically excited. Ultrasound energy reflects from tissue, and part of it returns to the transducer, producing an electrical impulse which is converted into the image. Two major different transducer designs exist: (a) the mechanically single rotating transducer and (b) the electronic system using an annular array of small crystals. Current IVUS systems operate at frequencies between 20 and 60 MHz. A higher frequency allows higher resolution but at the cost of lower depth penetration. This drawback has been limited by improvements in contemporary transducer designs. A high-definition IVUS system allows rapid image acquisition, faster catheter pullback speed, and superior axial resolution compared with conventional IVUS. Automatic motorized pullback is strongly recommended for acquiring high-quality IVUS images. The automatic pullback speed ranges from 0.5 to 10 mm/s in current IVUS systems [4].

Optical coherence tomography (OCT) is an invasive imaging modality that uses a catheter-based system. It includes a rotating single fiberoptic wire, paired with an imaging lens, all housed within a short monorail sheath. This setup enables the emission and recording of light reflection. Images are acquired during an automated pullback along the coronary vessels. The light source directs near-infrared light at the artery wall, which is then back-scattered to the catheter and analyzed by a technique called low-coherence interferometry. This facilitates the acquisition of high-resolution, cross-sectional images of the arterial wall, enabling the generation of three-dimensional volumetric reconstructions for advanced morphological and quantitative analysis [5]. Due to the fact that the OCT signal is scattered by blood, OCT image acquisition requires flushing in order to displace blood. OCT systems use shorter wavelengths at the spectrum of the infrared light (ranging from 1250 to 1350 nm) than the corresponding sound wavelengths that are used by IVUS (~25–80 μm, depending on frequency). Moreover, OCT achieves 10 times higher axial (10–20 mm) and lateral (20–90 mm) resolution than IVUS, leading to lower penetration depth (1–2 mm for OCT versus 5–6 mm for IVUS). Thus, OCT does not always allow visualization of the entire artery wall and complete plaque burden assessment. In contrast, plaque composition can be described precisely, enabling detection of superficial morphological characteristics that have been associated with high future risk of acute coronary syndrome [6].

Several randomized trials have been conducted to investigate the potential superiority of these two IVI modalities against classical coronary angiography in percutaneous coronary intervention (PCI) guidance and optimization with regard to either clinical or surrogate endpoints. Data referring to a head-to-head comparison of the two invasive imaging modalities are more limited. The IVUS-XPL trial demonstrated advantageous use of IVUS-guided long lesion PCI in terms of cardiovascular events driven by a reduction of ischemia-driven target-lesion revascularization [7]. The ULTIMATE trial amplified these conclusions since lower rates of clinically driven target-vessel failure at 12-month follow-up were achieved [8]. The OCTACS trial demonstrated more complete strut coverage when OCT was used to guide PCI with newer-generation DESs in patients admitted for acute coronary syndrome [9]. Similarly, the DOCTORS study linked OCT utilization with improved post-procedural fractional flow reserve in myocardial infarction patients [10]. The DETECT study evaluated OCT in patients with stable coronary artery disease and exhibited similar outcomes with trials that included unstable patients. In the ILUMIEN III study, OCT-guided PCI was non-inferior to IVUS, but also to angiography [11].

Our review aims to present and summarize all the latest available evidence and current status reports on intravascular imaging published since 2022. The outcomes of the recent randomized trials and main meta-analyses are described below and summarized in Table 1 and Table 3, respectively, while the methodological strengths and limitations of the main trials are summarized in Table 2.

### 1.2. Intravascular Imaging Versus Angiography

Until recently, evidence comparing intravascular imaging (IVI) with angiographic guidance in complex percutaneous coronary intervention (PCI) was limited. However, a landmark study by the RENOVATE-COMPLEX PCI investigators has significantly advanced this field [12]. The RENOVATE-COMPLEX PCI trial was a prospective, multicenter, randomized, open-label study conducted at 20 centers in South Korea. It enrolled 1639 patients with complex coronary artery disease, defined by the presence of unprotected left main disease, bifurcation lesions, severe calcification, in-stent restenosis, chronic total occlusion, ostial lesions, stent length ≥38 mm, or multivessel/multi-stent PCI. Participants were randomized in a 2:1 ratio to undergo either IVI-guided or angiography-guided PCI. Unlike the ILUMIEN IV and OCTOBER trials, both IVUS and OCT were permitted in the IVI arm.

At a median follow-up of 2.1 years, IVI guidance significantly reduced the primary endpoint of target-vessel failure compared with angiography (7.7% vs. 12.3%; hazard ratio, 0.64; 95% CI, 0.45–0.89; *p* = 0.008). The benefit of IVI was consistent across all predefined complex lesion subtypes, and no interaction was observed between the type of imaging modality used. However, it is noteworthy that only a minority of patients in the IVI group were treated using OCT, limiting direct comparisons between IVUS and OCT. There were no significant differences in procedural safety outcomes between the two groups. The incidence of cardiac death (1.7% vs. 3.8%), target-vessel myocardial infarction (3.7% vs. 5.6%), and repeat revascularization (6.3% vs. 5.6%) was comparable. A key limitation of the study is its single-country enrollment, as all participants were Korean, which may affect the generalizability of the findings to other populations [12].

A subanalysis of the RENOVATE-COMPLEX PCI trial involving 1639 patients with or without chronic kidney disease (CKD) suggested a consistent benefit of intravascular imaging-guided PCI of complex lesions regardless of the patient’s renal function status. Intravascular imaging-guided PCI was associated with lower incidence of target-vessel failure (consisting of a composite of cardiac mortality, myocardial infarction, or target-vessel revascularization compared to angiography-guided PCI, regardless of the patient’s renal function. The benefit of intravascular imaging regarding the primary endpoint was predominantly affected by the lower incidence of cardiac death or target-vessel-related myocardial infarction in patients with CKD. The greatest benefit of intravascular imaging guidance for TVF during complex PCI was shown in patients with stage 3 CKD (30 ≤ GFR < 60 mL/min/1.73 m^2^). This substudy demonstrated that CKD did not significantly alter the prognosis of complex PCI when guided by intravascular imaging, highlighting its beneficial role in complex revascularization even in CKD patients—a group characterized by high cardiovascular morbidity. However, it should be mentioned that a disproportionate utilization of intravascular imaging modalities led to a limited representation of OCT, thereby hindering a robust comparison of its benefits and risks relative to IVUS or angiography alone [13].

During the ESC Congress 2023, two randomized clinical trials were presented—OCTOBER and ILUMIEN IV—both evaluating the role of OCT-guided PCI in high-risk patients with bifurcation and complex coronary lesions, respectively. The Optical Coherence Tomography Optimized Bifurcation Event Reduction (**OCTOBER**) trial was a randomized, multicenter study designed to assess whether OCT-guided PCI is superior to angiography-guided PCI for the treatment of complex coronary bifurcation lesions. A total of 600 patients were randomized to OCT-guided PCI and 601 to angiography-guided PCI. Approximately one-fifth of patients (18.5% in the OCT group and 19.3% in the angiography group) had a bifurcation lesion involving the left main coronary artery. A two-stent strategy was used in 65% of patients.

At two-year follow-up, OCT guidance was superior to angiography in reducing the primary composite endpoint of major adverse cardiac events (MACEs)—defined as cardiac death, target-lesion myocardial infarction, and ischemia-driven target-lesion revascularization. MACEs occurred in 10.1% of patients in the OCT-guided group compared with 14.1% in the angiography-guided group (hazard ratio, 0.70; 95% confidence interval (CI), 0.50 to 0.98; *p* = 0.035). No significant differences in procedural complications were observed between groups.

Importantly, the clinical benefit of OCT-guided bifurcation PCI was particularly pronounced in patients treated with a single-stent strategy. Subgroup analysis also highlighted that left main angioplasty emerged as an independent predictor of favorable clinical outcomes when guided by OCT. Overall, OCT use in left main angioplasty was associated with improved clinical outcomes compared with angiography guidance. Although OCT guidance demonstrated a favorable safety profile, it was associated with longer procedural times and higher contrast volume [14]. OCTOBER was a sufficiently powered study showing for the first time that routine OCT guidance may improve clinical outcomes in a specific subset of lesions. The positive trial outcomes indicate that OCT facilitates stent optimization in a bifurcation stenting approach—a procedure defined traditionally by greater incidence of restenosis, stent thrombosis, and periprocedural MI [14].

The **ILUMIEN IV** trial enrolled 2500 patients randomized in a 1:1 ratio to undergo OCT-guided PCI or angiography-guided PCI. The ILUMIEN IV: OPTIMAL PCI trial was designed to evaluate the efficacy of OCT-guided PCI compared with angiography-guided PCI in patients with high-risk clinical profiles and/or complex coronary lesions. More specifically, the patients included were medication-treated patients with diabetes mellitus or presented with high-risk coronary artery lesions. The patients were followed for 2 years. A high-risk lesion was defined as severe calcification, diffuse or multi-focal in-stent restenosis, bifurcation lesion with planned two-stent approach, increased length (≥28 mm), chronic total occlusion (CTO), a non-ST elevation myocardial infarction (NSTEMI), or a delayed ST-segment elevation myocardial infarction (STEMI) (>24 h from symptom onset). Regarding the primary clinical endpoint defined by a composite of cardiac death, ischemia-driven target-vessel revascularization, or target-vessel MI, a statistically significant difference was not observed. Despite the fact that ILUMIEN IV failed to meet to its primary endpoint with respect to the primary clinical endpoint, OCT-guided revascularization showed favorable outcomes regarding the primary imaging endpoint. The minimum stent area (MSA) following PCI was 5.72 ± 2.04 mm^2^ in the OCT arm and 5.36 ± 1.87 mm^2^ in the angiography arm (mean difference, 0.36 mm2; 95% confidence interval (CI), 0.21 to 0.51; *p* < 0.001). Furthermore, OCT-guided PCI was associated with a significantly reduced rate of stent thrombosis at the two-year follow-up, with an incidence of 0.5% versus 1.4% in the angiography-guided cohort, yielding a relative risk reduction of approximately 64% (HR = 0.36; 95% CI, 0.14–0.91; *p* = 0.02). Although the ILUMIEN IV was a negative study, the attainment of a larger minimal stent area—which is a predictor of major adverse cardiac events—suggests that OCT guidance may be beneficial in complex lesion PCI. A significant limitation of the study that could potentially influence the study’s results is the enrollment and follow-up of patients during the COVID-19 pandemic disease, which justifies the low frequency of repeat revascularization in both groups due to a significantly decreased number of elective interventions [15].

The investigators of the ILUMIEN IV trial published a substudy analyzing OCT-guided versus angiography-guided PCI outcomes in patients with complex angiographic lesions. The principal key messages provided by the substudy were, firstly, the significantly larger minimal stent area (MSA) in OCT-guided PCI compared with angiography-guided PCI (5.56 ± 1.95 mm^2^ vs. 5.26 ± 1.81 mm^2^, *p* < 0.001), similarly to what was observed in the main study, highlighting again the OCT use is associated with better stent optimization, potentially improving vessel patency and reducing complications like restenosis. Unlike the main study, where no significant difference was found in clinical endpoints between OCT guidance and angiographic guidance, in the subset of complex lesions, OCT-guided PCI showed a lower risk of serious MACEs (including cardiac death, target-vessel myocardial infarction, or stent thrombosis) compared with angiography-guided PCI, lending further evidence to the hypothesis that the main benefit of intravascular imaging use is derived by complex lesion subsets and not solely by the presence of high-risk clinical features. Although OCT guidance improved stent expansion and the clinical endpoint of serious MACEs, there was no significant difference in target-vessel failure (TVF) between the OCT-guided and angiography-guided groups, indicating that, while OCT guidance can improve certain important clinical outcomes, its impact in endpoints such as repeat revascularization may be less pronounced [16].

While both the ILUMIEN IV and OCTOBER trials evaluated the role of OCT-guided percutaneous coronary intervention (PCI) in complex coronary artery disease, their outcomes diverged significantly. ILUMIEN IV, despite demonstrating improved stent optimization, failed to achieve its primary clinical endpoint—an outcome potentially attributable to differences in patient selection criteria, endpoint definitions, or the pandemic-related decline in elective procedures during the COVID-19 era. In contrast, the OCTOBER trial reported a significant reduction in major adverse cardiac events, particularly in cases involving left main and bifurcation lesions managed with a single-stent strategy. This disparity may also reflect variations in procedural approaches and regional differences in operator expertise. Collectively, these findings suggest that the clinical utility of OCT may be most evident in specific lesion subsets—particularly bifurcations and left main disease—where its high-resolution imaging facilitates more precise procedural decision-making [14,15].

The benefits of OCT were further demonstrated in a recent Korean study published in 2024. According to the findings of the OCCUPI randomized trial, OCT was found to be superior to conventional angiographic guidance in patients with complex coronary artery disease undergoing PCI. Complex lesions were defined to include chronic total occlusions, long lesions, bifurcations, calcified vessels, unprotected left main disease, small vessels, and acute myocardial infarction (both STEMI and NSTEMI).

This superiority was primarily driven by a reduction in ischemia-driven target-vessel revascularization and spontaneous myocardial infarction at 12 months. These clinical benefits were likely attributable to improved stent optimization in the OCT-guided group, which achieved a larger final stent diameter (3.2 mm vs. 3.0 mm, *p* < 0.001) and a greater minimal post-procedural lumen diameter (2.75 mm vs. 2.59 mm, *p* < 0.001).

Notably, within the OCT group, patients who achieved optimal stent expansion—defined as acceptable expansion, apposition, and absence of edge dissection—had a significantly lower incidence of the primary composite endpoint (cardiac death, myocardial infarction, stent thrombosis, or ischemia-driven target-vessel revascularization), with a hazard ratio of 0.33 (95% CI, 0.17–0.65; *p* = 0.0012). These findings underscore the importance of operator expertise, the use of standardized imaging protocols, and potentially adjunctive technologies in maximizing the benefits of OCT.

Importantly, although contrast volume was higher in the OCT group, no associated increase in contrast-induced nephropathy was observed, reaffirming the safety profile of the modality [17].

### 1.3. IVUS Versus OCT

The OCTIVUS trial was another recent study conducted in South Korea, designed similarly to the RENOVATE-COMPLEX PCI trial. This prospective, multicenter, investigator-initiated, open-label trial enrolled 2008 patients with significant coronary artery lesions undergoing PCI, who were randomized in a 1:1 ratio to receive either IVUS-guided or OCT-guided PCI. The primary aim was a head-to-head comparison of the two intravascular imaging modalities for PCI guidance. Imaging in both groups was performed using either IVUS with a rotational transducer (Opticross or Opticross HD, Boston Scientific, San Jose, CA, USA) or OCT (C7-XR and OPTIS, Abbott, Santa Clara, CA, USA) at baseline, during PCI, and post-stent deployment. Unlike the OCTOBER and ILUMIEN IV trials, which focused on complex lesions, OCTIVUS included a broad spectrum of coronary artery lesions, following diagnostic coronary angiography. The study aimed to demonstrate the non-inferiority of OCT compared with IVUS for the primary endpoint: a composite of cardiovascular death, target-vessel myocardial infarction, or target-vessel revascularization at one year. At one-year follow-up, the trial successfully met its non-inferiority criteria: the primary endpoint occurred in 2.5% of the OCT-guided group and 3.1% of the IVUS-guided group (*p* < 0.001 for non-inferiority). There were no significant differences in safety outcomes, including contrast-induced nephropathy (1.4% for OCT vs. 1.5% for IVUS), despite higher contrast volume use in the OCT arm. Notably, the procedure duration was shorter in the OCT group. Although IVUS-guided PCI achieved slightly better stent expansion, this did not translate into superior clinical outcomes. Interestingly, major procedural complications were fewer in the OCT group (2.2%) compared with the IVUS group (3.7%). The OCTIVUS trial adds valuable evidence to the existing literature, demonstrating that OCT and IVUS provide equivalent and comparable efficacy in PCI guidance. Moreover, both modalities exhibited favorable safety profiles, reinforcing their clinical utility in routine practice [18].

### 1.4. QCA- Versus IVUS-Guided PCI

The **GUIDE-DES** trial was a multicenter recently published trial performed in South Korea. Although it is not the first randomized trial that compares angiography-guided PCI versus IVUS-guided PCI, its novelty is based on the objective implementation of quantitative coronary angiography instead of a solely visual estimation of coronary lumen in the angiography arm. Adjusted QCA was calculated as measured QCA + 10% for vessels ≤3.5 mm, decreasing by 1% for every mm up to 4 mm; in vessels ≥4 mm, adjusted QCA was calculated as measured QCA + 5%. A total of 763 patients were enrolled in QCA-guided PCI and 765 patients enrolled in IVUS-guided PCI. The majority of participants were men with stable coronary disease, while half of them had multivessel disease. The study met its primary endpoint, which was non-inferiority with respect to target-lesion failure—defined as a composite of cardiac death, target-vessel myocardial infarction, or ischemia-driven target-lesion revascularization—at the first year (3.81% in the QCA-guided group versus 3.80% in the IVUS-guided group; *p* = 0.0002). No difference in stent thrombosis rates was observed between the two groups. GUIDE-DES is the first study that supports that utilization of QCA-angiography is non-inferior to intravascular imaging. However, more evidence is anticipated in the future to enrich our understanding about the comparability of the two modalities and confirm the above findings [19].

### 1.5. IVUS- Compared with FFR-Guided PCI in Intermediate-Severity Coronary Lesions

Fractional flow reserve (FFR) and IVUS are frequently used adjunctive invasive modalities that facilitate direct assessment of intermediate-degree coronary artery stenoses and aid decision-making for potential revascularization. The **FLAVOUR** trial was a head-to-head comparison of a strategy based on coronary anatomy, as assessed by IVUS, and a strategy based on coronary physiology, as assessed by FFR. A total of 1682 patients with intermediate-severity coronary disease (40% to 70% diameter stenosis of a coronary vessel estimated by angiography) were randomized. The follow-up duration was 2 years. The criteria to proceed to PCI included an FFR ≤ 0.80 for the 838 patients assigned to the physiology-guided group and a minimal lumen area (MLA) ≤ 3 mm^2^, or MLA > 3 but ≤4 mm^2^ plus plaque burden > 70%, for the 844 patients assigned to the IVUS group. Despite the established prognostic importance of FFR in chronic coronary syndromes, the study was designed based on the paradoxical hypothesis that the FFR-based strategy was non-inferior to the IVUS-based strategy. IVUS-guided PCI was performed in a greater number of cases in the IVUS group (65.3%) than in the FFR group (44.4%). Optimal PCI was achieved in a similar percentage in both groups (50.1% versus 54.8% in the FFR and the IVUS group, respectively). Non-inferiority was shown for FFR with regard to the primary endpoint, which was a composite of death, myocardial infarction, or revascularization. The primary endpoint was met in 8.1% of the enrolled patients in the FFR arm and in 8.5% of those in the IVUS arm (*p* = 0.01 for non-inferiority). FFR-guided PCI led to reduced target-vessel PCI compared with the IVUS-guided PCI. Fewer stents were implanted in the FFR group, and consequently, dual antiplatelet treatment was adopted more frequently in the IVUS group of patients. The results of the FLAVOUR trial suggest that both IVUS and FFR are equivalent and applicable tools that may aid the interventionalist in determining the severity of intermediate lesions [20].

### 1.6. Intravascular Imaging in Acute Coronary Syndromes

The role of intravascular imaging in acute coronary syndromes (ACSs) is evolving rapidly, supported by recent randomized evidence demonstrating its potential to improve procedural precision and clinical outcomes. Historically, the urgency and anatomical complexity associated with ACSs posed challenges for incorporating intravascular imaging into routine practice. However, recent data suggest that such imaging may be particularly beneficial in this high-risk population.

The **IVUS-ACS trial**, a large multicenter randomized study, significantly reshaped this paradigm by demonstrating that IVUS-guided PCI was superior to angiography-guided PCI in patients with ACSs. Specifically, the trial showed a 45% relative risk reduction in the composite endpoint of target-vessel failure at one year, driven by lower rates of myocardial infarction and repeat revascularization. These findings offer compelling support for expanding the use of IVUS beyond elective or stable settings, reinforcing its value even in urgent revascularizations where precise stent sizing and deployment are paramount [21].

Complementing this, the **OPINION ACS trial** directly compared two intravascular imaging modalities—optical frequency domain imaging (OFDI) and IVUS—in patients presenting with ACSs. OFDI was found to be non-inferior to IVUS in terms of stent expansion, as measured by the minimal post-procedural lumen area. This suggests that both modalities offer comparable mechanical optimization, allowing physicians to select the imaging strategy best suited to lesion morphology, operator expertise, and procedural logistics [22].

Together, these trials highlight a critical transition in ACS management: from reliance solely on angiography to a more nuanced, image-guided strategy. IVUS and OFDI not only enhance stent optimization but also contribute to improved risk stratification by identifying features such as plaque rupture, thrombus burden, and residual disease—elements that are often underestimated by angiography alone. These advances support the notion that intravascular imaging should not be reserved solely for complex or elective cases but rather become an integral part of modern PCI, including in the context of ACSs.

### 1.7. Meta-Analyses of Intravascular Imaging Guidance Versus Angiographic Guidance

**Figure 1:**
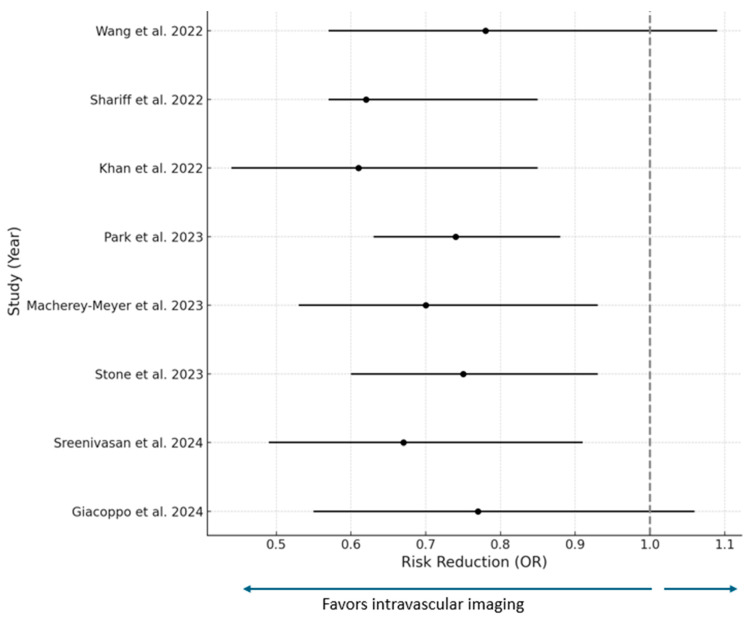
Summary of recent meta-analyses showing the superiority of IVI-guided PCI over angiography-guided PI for the endpoint of major adverse cardiovascular events [23,25,26,27,28,29,30,31].

Several recent meta-analyses have investigated the potential superiority of IVI-guided PCI over angiography-guided PCI (Table 3, Figure 1). A meta-analysis consisting of four RCTs and one observational study involving 3349 patients was the first meta-analysis that compared long-term clinical outcomes of IVUS- versus coronary angiography-guided PCI of long de novo coronary lesions with stent lengths over 28 mm. Utilization of IVUS in directing PCI with DESs led to a statistically significant decrease in long-term occurrence of the composite endpoint of MACEs (all myocardial infarctions, all vessel revascularizations, and stent thrombosis) during a median follow-up of 2 years (OR 0.41; 95% confidence interval (CI), 0.29–0.58; *p* < 0.00001), in consistency with previous meta-analyses. Myocardial infarction and revascularization rates were in favor of IVUS-guided PCI (OR 0.23; 95% CI, 0.09–0.58; *p* = 0.002 and OR 0.48; 95% CI, 0.36–0.66; *p* < 0.00001, respectively). However, no statistically significant difference was found in terms of all-cause mortality (OR 0.82; 95% CI, 0.55–1.23; *p* = 0.34) and stent thrombosis (OR 0.32; 95% CI, 0.11–0.89; *p* = 0.03) between IVUS-guided and angiography-guided PCI, in contrast with the meta-analysis by Zhang et al. [23,24]. A smaller number of patients undergoing IVUS-guided DES implantation could explain this discrepancy [23].

**Table 1:** List of recent randomized studies investigating the clinical impact of intravascular imaging guidance on PCI.

Study	Year	Enrolled (n)	Modality	Follow-Up (Months)	Primary Endpoint	Outcomes
RENOVATE-COMPLEX PCI [12]	2023	1639	IVUS vs. Angiography	25	TVF: Cardiac death, TV-MI, or TV revascularization	⮚ TVF: 7.7% (IVUS) vs. 12.3% (Angiography), *p* = 0.008. Stent thrombosis: 0.1% vs. 0.7%
OCTOBER [14]	2023	1201	OCT vs. Angiography	24	MACEs: Cardiac death, TL-MI, or TL revascularization	⮚ MACEs: 10.1% (OCT) vs. 14.1% (Angiography), *p* = 0.035. ⮚ Procedural complications: 6.8% vs. 5.7%
ILUMIEN IV [15]	2023	2487	OCT vs. Angiography	24	Minimum stent area (MSA) after PCI; Target-vessel failure (TVF)	⮚ MSA: 5.72 ± 2.04 mm^2^ (OCT) vs. 5.36 ± 1.87 mm^2^ (Angiography), *p* < 0.001. ⮚ No significant difference in TVF (7.4% vs. 8.2%, *p* = 0.45)
OCCUPI [17]	2024	1604	OCT vs. Angiography	12	MACEs: Cardiac death, MI, stent thrombosis, or ischemia-driven TVR	⮚ MACEs: 5% (OCT) vs. 7% (Angiography), *p* = 0.023. ⮚ No difference in CIN or mortality
OCTIVUS [18]	2023	2008	OCT vs. IVUS	12	Cardiac death, TV-MI, or TV revascularization	⮚ Non-inferiority met. Primary event: 2.5% (OCT) vs. 3.1% (IVUS), *p* < 0.001. ⮚ Major procedural complications: 2.2% vs. 3.7%, *p* = 0.04.
GUIDE-DES [19]	2024	1528	QCA-Angiography vs. IVUS	12	Target-lesion failure (TLF)	⮚ TLF: 3.81% (QCA) vs. 3.80% (IVUS), *p* = 0.99. ⮚ No difference in stent edge dissection, perforation, or thrombosis
FLAVOUR [20]	2022	1682	FFR vs. IVUS	24	Death, MI, or revascularization	⮚ Endpoint: 8.1% (FFR) vs. 8.5% (IVUS), *p* = 0.01 (non-inferiority)
IVUS-ACS [21]	2024	3504	IVUS vs. Angiography	12	TVF	⮚ TVF: 4.0% (IVUS) vs. 7.3% (Angiography), *p* = 0.0001. ⮚ Lower MI and TVR in IVUS group
OPINION ACS [22]	2024	158	OFDI vs. IVUS	12	Minimum stent area	⮚ Smaller minimum stent area for OFDI vs. IVUS (*p* = 0.096). ⮚ OFDI showed fewer proximal edge dissections (*p* = 0.012) and irregular protrusion (*p* = 0.03).

(FLAVOUR: Fractional Flow Reserve and Intravascular Ultrasound-Guided Intervention Strategy for Clinical Outcomes in Patients with Intermediate Stenosis, FFR: Fractional Flow Reserve, GUIDE-DES: Quantitative Coronary Angiography-Guidance versus Intravascular Ultrasound-Guidance for Drug-Eluting Stent Implantation, ILUMIEN IV: OCT-Guided Coronary Stent Implantation Compared with Angiography: A Multicenter Randomized Trial in PCI, OCT, or Angiography Guidance for PCI in Complex Bifurcation Lesions, IVUS: Intravascular Ultrasound, IVUS-ACSs: Intravascular Ultrasound-Guided versus Angiography-Guided Percutaneous Coronary Intervention in Acute Coronary Syndromes, OCCUPI: Optical Coherence Tomography-Guided Coronary Intervention in Patients With Complex Lesions, OCT: Optical Coherence Tomography, MSA: Minimum Stent Area, OCTIVUS: Optical Coherence Tomography versus Intravascular Ultrasound-Guided Percutaneous Coronary Intervention, OFDI: Optical Frequency Domain Imaging, OPINION ACSs: Optical Frequency Domain Imaging-Guided versus Intravascular Ultrasound-Guided Percutaneous Coronary Intervention for Acute Coronary Syndromes, QFR: Quantitative Flow Ratio, RENOVATE-COMPLEX PCI: Randomized Controlled Trial of Intravascular Imaging Guidance versus Angiography-Guidance on Clinical Outcomes After Complex Percutaneous Coronary Intervention).

**Table 2:** Methodological strengths and limitations of key trials.

Study Name	Key Strengths	Key Limitations
ILUMIEN IV [15]	Large RCT; high-risk lesion focus; imaging and clinical endpoints	Did not meet primary clinical endpoint; COVID-era biases
RENOVATE-COMPLEX PCI [12]	Included both IVUS and OCT; multicenter; large sample of complex lesions	Majority IVUS-guided; single-country population (Korea)
OCTOBER [14]	Specific to bifurcation and LMCA lesions; clear clinical benefit shown	Benefit mainly in single-stent strategy; limited generalizability
ILUMIEN IV [15]	Large, multicenter, randomized trial; direct comparison of OCT-guided vs. angiography-guided PCI; powered for clinical outcomes	No significant difference in primary endpoint; majority stable CAD patients
OCCUPI [17]	Strong procedural and optimization data; real-world complexity	Left main and small vessel subgroups underpowered
OCTIVUS [18]	Head-to-head comparison; broad lesion types; non-inferiority shown	No clear clinical outcome difference; procedural complication variance
GUIDE-DES [19]	First trial using QCA vs. IVUS; novel design	Short follow-up; mostly stable patients
FLAVOUR [20]	Head-to-head comparison of physiology vs. imaging-based strategies; long follow-up	Limited to intermediate lesions; not imaging modality vs. angiography
IVUS-ACS [21]	Large trial in ACSs; strong evidence for IVUS in urgent settings	Limited OCT comparison; ACS subset only
OPINION ACS [22]	Direct comparison of OFDI vs. IVUS; ACS specific	Small sample size; short follow-up

**Table 3:** List of recent meta-analyses investigating the clinical impact of intravascular imaging guidance on PCI.

Study	Study Population	All-Cause Mortality	Cardiovascular Mortality	MACEs	Myocardial Infarction	Stent Thrombosis	TVR	TLR
Wang et al., 2022 [23]	5 studies (4 RCTs and 1 observational 3349 patients	OR 0.74 (0.39–1.39)	OR 0.82 (0.55–1.23)	0.78 (0.57–1.09)	OR 0.41 (0.29–0.58)	OR 0.32 (0.11–0.89)	OR 0.48 (0.36–0.6)	N/R
Shariff et al., 2022 [25]	14 RCTs	OR 0.97 (0.70–0.35)	OR 1.97 (1.25–3.11)	OR 1.62 (1.17–2.24)	OR 1.18 (0.81–1.73)	N/R	OR 1.60 (1.21–2.13)	N/R
Khan et al. IVI vs. CA [26]	20 RCTs 11,698 patients	OR 0.81 (0.64–1.02)	OR 0.53 (0.39–0.72)	N/R	OR 0.81 (0.68–0.97)	OR 0.44 (0.27–0.72)	OR 0.74 (0.61–0.89)	OR 0.71 (0.59–0.86)
Park et al., 2023 IVUS vs. CA [27]	28 studies 12,895 patients	OR 1.15 (0.85–1.56)	OR 0.64 (0.43–0.94)	OR 0.74 (0.63–0.88)	OR 0.82 (0.64–1.04)	OR 0.61 (0.36–1.04)	OR 0.64 (0.5–0.81)	OR 0.68 (0.57–0.8)
Macherey-Meyer et al., 2023 OCT vs. ICA [28]	8 studies 2612 patients	OR (0.51–2.31)	OR 0.49 (0.250.96)	OR 0.7 (0.53–0.93)	OR 0.82 (0.49–1.37)	N/R	OR 0.54 (0.26–1.13)	OR 0.26 (0.07–0.95)
Stone et al., 2023 IVI vs. ICA [29]	22 studies 15,694 patients	OR 0.75 (0.6–0.93)	OR 0.55 (0.41–0.75)	N/R	OR 0.83 (0.71–0.99)	OR 0.52 (0.34–0.81)	OR 0.64 (0.38–1.07)	OR 0.72 (0.60–0.86)
Sreenivasan et al., 2024 IVI vs. ICA [30]	16 studies 7814 patients	OR 0.75 (0.55–1.02)	OR 0.49 (0.34–0.71)	OR 0.67 (0.55–0.82)	N/R	OR 0.63 0.40–0.99	OR 0.60 (0.45–0.80)	OR 0.67 (0.49–0.91)
Giacoppo et al., 2024 IVUS vs. OCT vs. ICA [31]	24 RCTs 15,489 patients • IVUS vs. ICA • OCT vs. ICA • OCT vs. IVUS	OR 0.77 (0.55–1.06) OR 0.71 (0.51–0.99) OR 0.93 (0.61–1.41)	OR 0.57 (0.37–0.90) OR 0.58 (0.69–0.94) OR 1.01 (0.55–1.84)	OR 0.67 (0.56–0.8) OR 0.77 (0.63–0.94) OR 1.14 (0.9–1.45)	OR 0.88 (0.67–1.17) OR 0.90 (0.70–1.16) OR 0.83 (0.44–1.56)	OR 0.6 (0.35–1.05) OR 0.49 (0.26–0.92) OR 0.81 (0.37–1.79)	OR 0.65 (0.53–0.81) OR 0.87 (0.68–1.11) OR 1.33 (1–1.77)	OR 0.69 (0.54–0.87) OR 0.83 (0.63–1.09) OR 1.2 (0.88–1.66)

(MACEs: Major Adverse Cardiovascular Events, TVR: Target-Vessel Revascularization, TLR: Target-Lesion Revascularization, OR: Odds Ratio).

The meta-analysis by Shariff et al. in 2022 [25] confirmed the superiority of intravascular imaging in guiding first- and second-generation DES implantation compared with angiography in terms of MACEs. A total of 14 randomized clinical trials were included. A larger number of major cardiovascular events (OR, 1.62; 95% CI, 1.17–2.24) and higher cardiovascular mortality (OR, 1.97; 95% CI, 1.25–3.11) were noted at the expense of the use of angiography. No difference was observed in the incidence of myocardial infarction and all-cause mortality. Target-vessel revascularization was more frequent when angiography was used instead of IVUS, but there was no difference for this endpoint between angiography and OCT. OCT was not proven inferior to IVUS; however, no robust conclusions can be derived regarding a head-to-head comparison between the two imaging modalities from this meta-analysis due to the limited amount of data incorporated in the included trials for this comparison [25].

A meta-analysis comprising 11,698 patients who underwent percutaneous coronary intervention with drug-eluting stents confirmed the superiority of intravascular imaging guidance over intracoronary angiography guidance. The study encouraged the use of intravascular imaging due to the achievement of a lower incidence of cardiac death and improved cardiovascular outcomes. However, this occurred at the cost of extended procedural and fluoroscopy duration. The protective role of intravascular imaging with respect to limiting cardiovascular events was more evident for lesions with a higher SYNTAX score [26].

A large meta-analysis involving 28 RCTs on IVUS-, OCT-, and coronary angiography-guided PCI, in agreement with the majority of the existing evidence, revealed the superiority of intravascular ultrasound over angiography for the endpoints of major adverse cardiovascular events, cardiovascular mortality, stent thrombosis, and target-lesion and target-vessel revascularization. Similar advantages were shown for OCT versus coronary angiography, but this benefit was evident only for PCI with DESs and interventions performed in patients with acute coronary syndromes. OCT and IVUS guidance had similar outcomes. Nonetheless, the low number of studies and patients with OCT-guided PCI did not allow for drawing safe conclusions about OCT [27].

Macherey-Meyer et al. conducted the first meta-analysis specifically designed to compare OCT-guided and angiography-guided PCI in patients with acute coronary syndrome. A 30% lower risk of major cardiovascular events and a 74% lower risk of target-lesion revascularization were demonstrated when OCT was used to guide PCI in these patients. Cardiac death was reduced by approximately one half, although no significant difference was observed in all-cause mortality [28].

The recently presented network meta-analysis by Stone et al. at the ESC Congress 2023 included data from the latest four randomized trials (ILUMIEN IV, OCTOBER, OCTIVUS, and RENOVATE-COMPLEX PCI). A notable difference compared with previous guidelines is that this meta-analysis was specifically designed to incorporate only randomized trials involving drug-eluting stents with longer follow-ups. A total of 15,964 patients enrolled in 22 trials were randomized to intravascular imaging guidance using OCT or IVUS versus angiography guidance. At a mean follow-up of 24.7 months, the intravascular imaging group demonstrated 29% lower odds of target-lesion failure, which was the primary endpoint of the study. This outcome was driven by a significant reduction in the components of the primary endpoint. Specifically, there was a 45% decrease in cardiovascular death, a 28% decrease in target-lesion revascularization, and an 18% decrease in target-vessel myocardial infarction. The incidence of definite or probable stent thrombosis was markedly reduced with intravascular imaging guidance by 48% compared with angiography guidance and by 61% in terms of definite stent thrombosis. Moreover, intravascular imaging-guided PCI led to a 25% reduction in all-cause death and a 17% reduction in myocardial infarction. Pooled analysis showed comparable outcomes between IVUS-guided PCI and OCT-guided PCI [29].

Another meta-analysis by Sreenivasan et al. suggests the adoption of intravascular imaging as a highly reliable strategy for PCI guidance. The study revealed a remarkable 33% reduction in major cardiovascular events in favor of intravascular imaging. Furthermore, cardiac death was reduced by approximately 50%. The clinical benefit of IVI was also supported by a 40% reduction in target-vessel revascularization, a 33% reduction in target-lesion revascularization, and a 39% reduction in target-vessel myocardial infarction. The authors noted that no inconsistency was observed among the 16 included randomized trials. However, the meta-analysis did not incorporate the latest published trials (ILUMIEN IV, OCTIVUS, and OCTOBER). Additionally, it should be highlighted that meta-regression analysis revealed a significant interaction between complex PCI and major adverse cardiovascular events (MACEs), suggesting that the complexity of the procedure may influence the association between intravascular imaging and clinical outcomes [30].

Giacoppo et al. presented a comprehensive and updated meta-analysis providing a detailed comparison between intravascular imaging-guided PCI and angiography-guided PCI. Data from 24 randomized trials involving 15,489 patients were analyzed. IVUS-guided PCI was compared with angiography-guided PCI in 46.4% of cases, OCT to angiography in 32.1%, and OCT to IVUS in 21.4% of patients. The network meta-analysis supports the use of intravascular imaging for PCI guidance, as it contributes to the reduction of any target-lesion revascularization and ischemia-driven target-lesion revascularization. These benefits were most prominent for IVUS, as no significant difference between OCT and angiography was observed. This finding was primarily attributed to the attenuated benefit observed in the ILUMIEN IV trial. Nevertheless, this meta-analysis demonstrated that OCT guidance was associated with a lower incidence of hard endpoints, specifically cardiac death and stent thrombosis [31].

## 2. Conclusions

Contemporary data obtained from lately published trials and numerous meta-analyses strongly support the use of intravascular imaging, particularly for PCI guidance in complex lesions. Previous guidelines, including European guidelines for coronary revascularization in 2018 and the corresponding of American Heart Association/American College of Cardiology in 2021, had upgraded the indication of intravascular imaging guidance from class IIb to class IIa [32,33]. However, the previously described trials have added to the totality of data on the subject that demonstrate a consistent benefit of the routine use of intravascular imaging in PCI guidance, thus motivating the authors of the latest ESC guidelines on Chronic Coronary Syndromes to recommend as a Class I indication the use of intravascular imaging when performing PCI on anatomically complex lesions—in particular, left main stem, true bifurcations, and long lesions [34]. The emerging evidence regarding the beneficial impact of intravascular imaging guidance establishes the need for a significant increase in clinical adaptation, with intravascular imaging becoming mandatory in complex lesions, as well as the need for more interventional cardiologists to be familiarized with intravascular imaging modalities and adopting them to their daily clinical practice. Therefore, interventional cardiologists need to take action that promotes increased utilization of intravascular imaging modalities and promote educational activities to help more interventional cardiologists incorporate them into their daily practice. Specifically, intravascular ultrasound (IVUS) is preferred in long lesions, heavily calcified vessels, and chronic total occlusions, where deep tissue penetration is essential. Optical coherence tomography (OCT), on the other hand, offers high-resolution imaging ideal for bifurcation lesions, left main interventions, and ACS cases with uncertain plaque morphology. Incorporating modality-specific strategies based on lesion anatomy can help maximize clinical outcomes in percutaneous coronary intervention.

## Data Availability

Not applicable. No new data were created or analyzed in this study. Data sharing is not applicable to this article.

## Abbreviations

The following abbreviations are used in this manuscript:

ACS	Acute Coronary Syndrome
FLAVOUR	Fractional Flow Reserve and Intravascular Ultrasound-Guided Intervention Strategy for Clinical Outcomes in Patients with Intermediate Stenosis
FFR	Fractional Flow Reserve
GUIDE-DES	Quantitative Coronary Angiography-Guidance versus Intravascular Ultrasound-Guidance for Drug-Eluting Stent Implantation
ILUMIEN IV	OCT-Guided Coronary Stent Implantation Compared with Angiography: A Multicenter Randomized Trial in PCI, OCT, or Angiography Guidance for PCI in Complex Bifurcation Lesions
IVUS	Intravascular Ultrasound
IVUS-ACSs	Intravascular Ultrasound-Guided versus Angiography-Guided Percutaneous Coronary Intervention in Acute Coronary Syndromes
MACEs	Major Adverse Cardiovascular Events
MSA	Minimum Stent Area
OCCUPI	Optical Coherence Tomography-Guided Coronary Intervention in Patients With Complex Lesions
OCT	Optical Coherence Tomography
OCTIVUS	Optical Coherence Tomography versus Intravascular Ultrasound-Guided Percutaneous Coronary Intervention
OFDI	Optical Frequency Domain Imaging
OPINION ACSs	Optical Frequency Domain Imaging-Guided versus Intravascular Ultrasound-Guided Percutaneous Coronary Intervention for Acute Coronary Syndromes
OR	Odds Ratio
QFR	Quantitative Flow Ratio
RENOVATE-COMPLEX PCI	Randomized Controlled Trial of Intravascular Imaging Guidance versus Angiography-Guidance on Clinical Outcomes After Complex Percutaneous Coronary Intervention
TLR	Target-Lesion Revascularization
TVR	Target-Vessel Revascularization

## References

[1] Mintz G.S., Guagliumi G. (2017). Intravascular imaging in coronary artery disease. Lancet.

[2] Mintz G.S., Popma J.J., Pichard A.D., Kent K.M., Satler L.F., Chuang Y.C., DeFalco R.A., Leon M.B. (1996). Limitations of angiography in the assessment of plaque distribution in coronary artery disease: A systematic study of target lesion eccentricity in 1446 lesions. Circulation.

[3] Mintz G.S. (2014). Clinical utility of intravascular imaging and physiology in coronary artery disease. J. Am. Coll. Cardiol..

[4] Saito Y., Kobayashi Y., Fujii K., Sonoda S., Tsujita K., Hibi K., Morino Y., Okura H., Ikari Y., Honye J. (2022). Clinical expert consensus document on intravascular ultrasound from the Japanese Association of Cardiovascular Intervention and Therapeutics (2021). Cardiovasc. Interv. Ther..

[5] Bezerra H.G., Costa M.A., Guagliumi G., Rollins A.M., Simon D.I. (2009). Intracoronary optical coherence tomography: A comprehensive review clinical and research applications. JACC Cardiovasc. Interv..

[6] Ali Z.A., Karimi Galougahi K., Mintz G.S., Maehara A., Shlofmitz R.A., Mattesini A. (2021). Intracoronary optical coherence tomography: State of the art and future directions. EuroIntervention.

[7] Hong S.J., Mintz G.S., Ahn C.M., Kim J.S., Kim B.K., Ko Y.G., Kang T.S., Kang W.C., Kim Y.H., Hur S.H. (2020). Effect of Intravascular Ultrasound-Guided Drug-Eluting Stent Implantation: 5-Year Follow-Up of the IVUS-XPL Randomized Trial. JACC Cardiovasc. Interv..

[8] Gao X.F., Ge Z., Kong X.Q., Kan J., Han L., Lu S., Tian N.L., Lin S., Lu Q.H., Wang X.Y. (2021). 3-Year Outcomes of the ULTIMATE Trial Comparing Intravascular Ultrasound Versus Angiography-Guided Drug-Eluting Stent Implantation. JACC Cardiovasc. Interv..

[9] Antonsen L., Thayssen P., Maehara A., Hansen H.S., Junker A., Veien K.T., Hansen K.N., Hougaard M., Mintz G.S., Jensen L.O. (2015). Optical Coherence Tomography Guided Percutaneous Coronary Intervention With Nobori Stent Implantation in Patients With Non-ST-Segment-Elevation Myocardial Infarction (OCTACS) Trial: Difference in Strut Coverage and Dynamic Malapposition Patterns at 6 Months. Circ. Cardiovasc. Interv..

[10] Meneveau N., Souteyrand G., Motreff P., Caussin C., Amabile N., Ohlmann P., Morel O., Lefrancois Y., Descotes-Genon V., Silvain J. (2016). Optical Coherence Tomography to Optimize Results of Percutaneous Coronary Intervention in Patients with Non-ST-Elevation Acute Coronary Syndrome: Results of the Multicenter, Randomized DOCTORS Study (Does Optical Coherence Tomography Optimize Results of Stenting). Circulation.

[11] Lee S.Y., Ahn C.M., Yoon H.J., Hur S.H., Kim J.S., Kim B.K., Ko Y.G., Choi D., Jang Y., Hong M.K. (2018). Early Follow-Up Optical Coherence Tomographic Findings of Significant Drug-Eluting Stent Malapposition. Circ. Cardiovasc. Interv..

[12] Lee J.M., Choi K.H., Song Y.B., Lee J.Y., Lee S.J., Lee S.Y., Kim S.M., Yun K.H., Cho J.Y., Kim C.J. (2023). Intravascular Imaging-Guided or Angiography-Guided Complex PCI. N. Engl. J. Med..

[13] Kwon W., Choi K.H., Song Y.B., Park Y.H., Lee J.M., Lee J.Y., Lee S.J., Lee S.Y., Kim S.M., Yun K.H. (2023). Intravascular Imaging in Patients With Complex Coronary Lesions and Chronic Kidney Disease. JAMA Netw. Open.

[14] Holm N.R., Andreasen L.N., Neghabat O., Laanmets P., Kumsars I., Bennett J., Olsen N.T., Odenstedt J., Hoffmann P., Dens J. (2023). OCT or Angiography Guidance for PCI in Complex Bifurcation Lesions. N. Engl. J. Med..

[15] Ali Z.A., Landmesser U., Maehara A., Matsumura M., Shlofmitz R.A., Guagliumi G., Price M.J., Hill J.M., Akasaka T., Prati F. (2023). Optical Coherence Tomography-Guided versus Angiography-Guided PCI. N. Engl. J. Med..

[16] Ali Z.A., Landmesser U., Maehara A., Shin D., Sakai K., Matsumura M., Shlofmitz R.A., Leistner D., Canova P., Alfonso F. (2024). OCT-Guided vs Angiography-Guided Coronary Stent Implantation in Complex Lesions: An ILUMIEN IV Substudy. J. Am. Coll. Cardiol..

[17] Hong S.J., Lee S.J., Lee S.H., Lee J.Y., Cho D.K., Kim J.W., Kim S.M., Hur S.H., Heo J.H., Jang J.Y. (2024). Optical coherence tomography-guided versus angiography-guided percutaneous coronary intervention for patients with complex lesions (OCCUPI): An investigator-initiated, multicentre, randomised, open-label, superiority trial in South Korea. Lancet.

[18] Kang D.Y., Ahn J.M., Yun S.C., Hur S.H., Cho Y.K., Lee C.H., Hong S.J., Lim S., Kim S.W., Won H. (2023). Optical Coherence Tomography-Guided or Intravascular Ultrasound-Guided Percutaneous Coronary Intervention: The OCTIVUS Randomized Clinical Trial. Circulation.

[19] Lee P.H., Hong S.J., Kim H.S., Yoon Y.W., Lee J.Y., Oh S.J., Lee J.S., Kang S.J., Kim Y.H., Park S.W. (2024). Quantitative Coronary Angiography vs Intravascular Ultrasonography to Guide Drug-Eluting Stent Implantation: A Randomized Clinical Trial. JAMA Cardiol..

[20] Yang S., Kang J., Hwang D., Zhang J., Jiang J., Hu X., Hahn J.Y., Nam C.W., Doh J.H., Lee B.K. (2024). Physiology- or Imaging-Guided Strategies for Intermediate Coronary Stenosis. JAMA Netw. Open.

[21] Li X., Ge Z., Kan J., Anjum M., Xie P., Chen X., Khan H.S., Guo X., Saghir T., Chen J. (2024). Intravascular ultrasound-guided versus angiography-guided percutaneous coronary intervention in acute coronary syndromes (IVUS-ACS): A two-stage, multicentre, randomised trial. Lancet.

[22] Otake H., Kubo T., Hibi K., Natsumeda M., Ishida M., Kataoka T., Takaya T., Iwasaki M., Sonoda S., Shinke T. (2024). Optical frequency domain imaging-guided versus intravascular ultrasound-guided percutaneous coronary intervention for acute coronary syndromes: The OPINION ACS randomised trial. EuroIntervention.

[23] Wang S., Liang C., Wang Y., Sun S., Wang Y., Suo M., Ye M., Li X., Liu X., Zhang M. (2022). The long-term clinical outcomes of intravascular ultrasound-guided versus angiography-guided coronary drug eluting stent implantation in long de novo coronary lesions: A systematic review and meta-analysis. Front. Cardiovasc. Med..

[24] Zhang Y., Farooq V., Garcia-Garcia H.M., Bourantas C.V., Tian N., Dong S., Li M., Yang S., Serruys P.W., Chen S.L. (2012). Comparison of intravascular ultrasound versus angiography-guided drug-eluting stent implantation: A meta-analysis of one randomised trial and ten observational studies involving 19,619 patients. EuroIntervention.

[25] Shariff M., Kumar A., Kansara T., Majmundar M., Doshi R., Stulak J.M., Kapadia S.R., Reed G.W., Puri R., Kalra A. (2022). Network Meta-analysis of Trials Comparing Intravascular Ultrasound, Optical Coherence Tomography, and Angiography-Guided Technique for Drug-Eluting Stent Implantation. J. Soc. Cardiovasc. Angiogr. Interv..

[26] Khan S.U., Agarwal S., Arshad H.B., Akbar U.A., Mamas M.A., Arora S., Baber U., Goel S.S., Kleiman N.S., Shah A.R. (2023). Intravascular imaging guided versus coronary angiography guided percutaneous coronary intervention: Systematic review and meta-analysis. BMJ.

[27] Park D.Y., An S., Jolly N., Attanasio S., Yadav N., Gutierrez J.A., Nanna M.G., Rao S.V., Vij A. (2023). Comparison of intravascular ultrasound, optical coherence tomography, and conventional angiography-guided percutaneous coronary interventions: A systematic review, network meta-analysis, and meta-regression. Catheter. Cardiovasc. Interv..

[28] Macherey-Meyer S., Meertens M.M., Heyne S., Braumann S., Tichelbacker T., Wienemann H., Mauri V., Baldus S., Adler C., Lee S. (2024). Optical coherence tomography-guided versus angiography-guided percutaneous coronary intervention in acute coronary syndrome: A meta-analysis. Clin. Res. Cardiol..

[29] Stone G.W., Christiansen E.H., Ali Z.A., Andreasen L.N., Maehara A., Ahmad Y., Landmesser U., Holm N.R. (2024). Intravascular imaging-guided coronary drug-eluting stent implantation: An updated network meta-analysis. Lancet.

[30] Sreenivasan J., Reddy R.K., Jamil Y., Malik A., Chamie D., Howard J.P., Nanna M.G., Mintz G.S., Maehara A., Ali Z.A. (2024). Intravascular Imaging-Guided Versus Angiography-Guided Percutaneous Coronary Intervention: A Systematic Review and Meta-Analysis of Randomized Trials. J. Am. Heart Assoc..

[31] Giacoppo D., Laudani C., Occhipinti G., Spagnolo M., Greco A., Rochira C., Agnello F., Landolina D., Mauro M.S., Finocchiaro S. (2024). Coronary Angiography, Intravascular Ultrasound, and Optical Coherence Tomography for Guiding of Percutaneous Coronary Intervention: A Systematic Review and Network Meta-Analysis. Circulation.

[32] Neumann F.J., Sousa-Uva M., Ahlsson A., Alfonso F., Banning A.P., Benedetto U., Byrne R.A., Collet J.P., Falk V., Head S.J. (2019). 2018 ESC/EACTS Guidelines on myocardial revascularization. EuroIntervention.

[33] Lawton J.S., Tamis-Holland J.E., Bangalore S., Bates E.R., Beckie T.M., Bischoff J.M., Bittl J.A., Cohen M.G., DiMaio J.M., Don C.W. (2022). 2021 ACC/AHA/SCAI Guideline for Coronary Artery Revascularization: Executive Summary: A Report of the American College of Cardiology/American Heart Association Joint Committee on Clinical Practice Guidelines. Circulation.

[34] Vrints C., Andreotti F., Koskinas K.C., Rossello X., Adamo M., Ainslie J., Banning A.P., Budaj A., Buechel R.R., Chiariello G.A. (2024). 2024 ESC Guidelines for the management of chronic coronary syndromes. G. Ital. Cardiol..

